# The Evolutionary Relevance of Social Learning and Transmission in Non-Social Arthropods with a Focus on Oviposition-Related Behaviors

**DOI:** 10.3390/genes12101466

**Published:** 2021-09-22

**Authors:** Caroline M. Nieberding, Matteo Marcantonio, Raluca Voda, Thomas Enriquez, Bertanne Visser

**Affiliations:** 1 Evolutionary Ecology and Genetics Group, Earth and Life Institute, UCLouvain, 1348 Louvain-la-Neuve, Belgium; matteo.marcantonio@uclouvain.be (M.M.); raluca.voda@uclouvain.be (R.V.); 2 Evolution and Ecophysiology Group, Earth and Life Institute, UCLouvain, 1348 Louvain-la-Neuve, Belgium; thomas.enriquez@uclouvain.be (T.E.); bertanne.visser@uclouvain.be (B.V.)

**Keywords:** behavioral plasticity, communication, culture, *Drosophila*, fitness, herbivores, oviposition site selection, natural selection

## Abstract

Research on social learning has centered around vertebrates, but evidence is accumulating that small-brained, non-social arthropods also learn from others. Social learning can lead to social inheritance when socially acquired behaviors are transmitted to subsequent generations. Using oviposition site selection, a critical behavior for most arthropods, as an example, we first highlight the complementarities between social and classical genetic inheritance. We then discuss the relevance of studying social learning and transmission in non-social arthropods and document known cases in the literature, including examples of social learning from con- and hetero-specifics. We further highlight under which conditions social learning can be adaptive or not. We conclude that non-social arthropods and the study of oviposition behavior offer unparalleled opportunities to unravel the importance of social learning and inheritance for animal evolution.

## 1. Introduction

**Figure 1:**
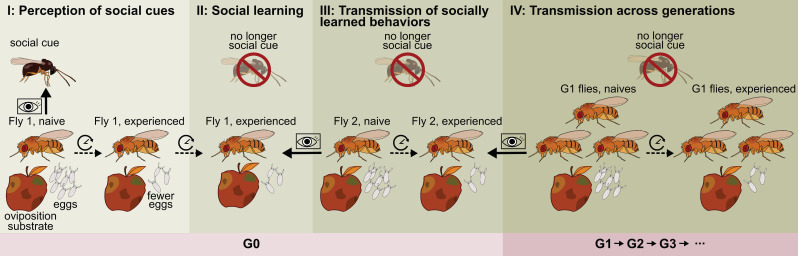
**The steps involved in social inheritance. Step I** Perception of social cues: Fly 1 perceives a social cue, e.g., the presence of a parasitic wasp that can parasitize and kill the larvae of *Drosophila melanogaster* (based on [21]). In response to the social cue, fly 1 changes its behavior, e.g., the female *D. melanogaster* reduces oviposition (fewer eggs are laid). The behavioral change proves that the cue is perceived. **Step II** Social learning: Fly 1 has learned about the social cue and is now experienced, meaning that the behavioral adjustment persists in time even when the social cue is no longer present, e.g., *D. melanogaster* females continue laying fewer eggs even when the wasp has left the patch. **Step III** Transmission: The socially learned behavior is taken over by naive fly 2 from experienced fly 1 (i.e., through visual and olfactory cues) that then changes its behavior. **Step IV** Transmission across generations: The socially learned behavior spreads throughout the population and over subsequent generations, e.g., other *Drosophila* females (including those belonging to other species) perceive the behavioral change of individuals 1 or 2 and also reduce their egg numbers (based on [22]). For social inheritance, naive flies belonging to the next generation should acquire behaviors from experienced flies exhibiting socially learned behaviors. This remains to be tested explicitly in the example of social learning of wasp threats in *Drosophila.* Of note, social inheritance can produce culture, based on additional criteria for transmission of socially learned behaviors as described in [23].

The emergence and spread of novel behaviors through social learning, or “learning from others”, has been documented in a wide variety of animals, mainly in social vertebrates [1,2,3,4,5]. In recent years, social learning has been demonstrated to act as the “second inheritance system”, called “social inheritance”, that functions in parallel with classical genetic inheritance in a number of social vertebrates in the wild. Social inheritance entails the perception of behaviors performed by others that are subsequently taken over (e.g., by imitation, imprinting or teaching) and spread throughout a population and subsequent generations [6,7,8,9] (see Figure 1 depicting the steps leading to social inheritance). Aside from examples in humans, remarkable evidence for cultural evolution includes the transmission of tool use in apes and song communication in birds and whales [8,10,11,12,13,14]. 

Social vertebrates have been at the forefront of research on social learning, but studies using small-brained and short-lived social invertebrates are increasing in number. In an exceptional experiment with *Bombus terrestris* bumblebees, Alem et al. [15] showed that some individuals can innovate by acquiring a non-natural, novel behavior for feeding: string pulling. Once demonstrator individuals (previously trained to pull a string to reach a sugar source) were observed by unexperienced individuals, these bees learned how to perform string pulling themselves. The authors further showed that string pulling behavior could spread from a single experienced individual (i.e., that perceived a social cue leading to a behavioral change) to other bees, even when the original demonstrator was no longer present (completing step 1 to 4 that demonstrate social inheritance as depicted in Figure 1; [15,16]). For invertebrates, most work has been done with social insects and recent findings support the idea that insects have the cognitive abilities necessary for transmission of socially learned behaviors [17,18,19,20].

In an intricate study by Danchin et al. [23], the authors use the fly *D. melanogaster* to show that social inheritance (producing basic traditions or culture) can arise and spread throughout subsequent generations. Female *D. melanogaster* made similar mate choice decisions as the female fly they observed earlier when offered a choice between males with contrasting phenotypes (colored pink or green) themselves. Transmission of color-based mate preference also occurred when younger females observed older females, meaning that the acquired preference could spread to a potential future generation as a tradition (i.e., step 4 in Figure 1). The authors further showed that long-term memory was involved, that mate preferences can be transferred repeatedly over time, and that conformism was involved (i.e., taking over the most common behavior), leading to a stable, cultural, mate choice preference in the population. This study provides a rare example of social inheritance in non-social insects (but see [24] that consider *D. melanogaster* as moderately social; and [22,25,26] provide evidence for transmission of socially learned behaviors, step 3 in Figure 1). While the potential fitness advantages of mate-copying are clear [27,28], pink and green males do not occur in nature, meaning that there is no ecological relevance and adaptive value of the artificial cue used in this study [29]. 

Social inheritance may play an important role in the evolution of non-social arthropods. In this perspective, we discuss relevant examples of social learning in the context of oviposition and related behaviors to illustrate the taxonomic diversity of observations in non-social arthropods. We also highlight why studying non-social arthropods is both relevant and timely. While learning of foraging, mating, host finding and other behaviors have been discussed elsewhere [30,31,32,33,34], here we focus on the social transmission of oviposition site selection. Oviposition site selection is a behavioral trait of key ecological significance for the relationship between organisms and their habitat, as the decision on where to lay eggs can have massive consequences for fitness and demography ([35] and references therein). This is particularly true for herbivorous arthropods with limited mobility as juveniles, because the egg-laying site is often also the offspring’s food source. Oviposition is a critical behavior with which colonization of new suitable habitats is initiated [35]. We start our perspective by illustrating the complementarities between genetic and social heredity using the hypothetical example of oviposition site selection. Next, we show that social learning related to oviposition is reported by an increasing number of studies (Table 1), adding to the ample evidence for non-social learning (i.e., learning solely from previous experience, or “autonomous” learning) for oviposition in wasps, flies, moths and butterflies (e.g., [36,37,38,39,40,41,42,43,44]). We then extend our discussion to cases where social learning of oviposition-related behaviors occurs not only from interactions between con-specifics, but also from hetero-specifics. Finally, we are paying particular attention to the evidence for, and quantification of, the adaptive value of social learning using existing empirical evidence for fitness effects.

## 2. Genetics, Epigenetics and Social Inheritance in the Context of Oviposition Site Selection

**Figure 2:**
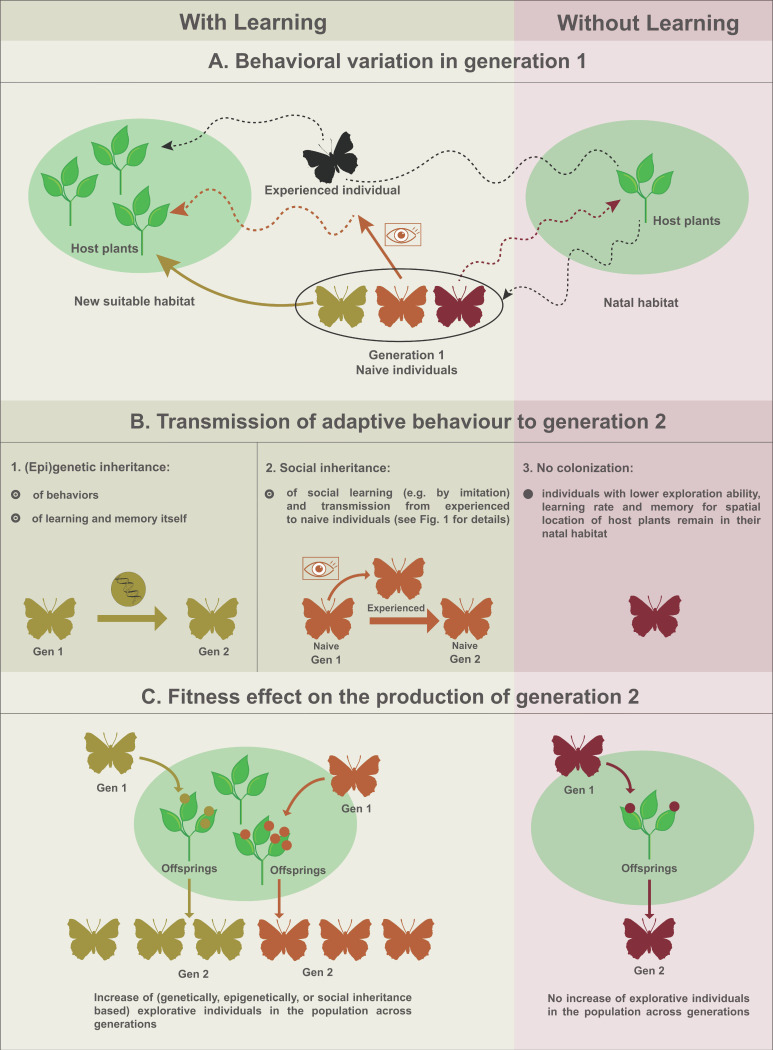
(Epi)genetic and social inheritance for oviposition site selection can affect the colonization of new suitable habitats with better host plant resources. **A**: Variation between individuals in oviposition site selection on host plants can be due either to (epi)genetic variation or variation in social learning skills. Social learning can lead to the colonization of new suitable habitats by naive individuals, for example by following experienced individuals towards a new habitat patch. Here, social learning is based on imitation and can occur through horizontal, oblique or (more rarely so) vertical transmission. Individuals not relying on social learning from conspecifics have a lower probability of finding new suitable habitats for oviposition. **B**: More adaptive behavioral variants for finding a new suitable habitat for oviposition can be transmitted through genetic or (epi)genetic variants (1). Transmission of social learning ability from parents to offspring can be genetically based or (epi)genetically transmitted. In addition, social learners outperform individuals not using social cues to learn about resource distribution in their environment (2). Social inheritance allows younger individuals to locate new habitats based on social information provided by older conspecifics. When there is no (epi)genetic basis for exploration, and learning and social learning does not occur, individuals have a lower probability of colonizing new habitats (3). **C**: The increasing ability of individuals within a population to learn and remember the spatial location of resources, such as host plants for oviposition, can be due to selection of (epi)genetic variants of the adaptive behavior, including learning rate and memory retention, or due to social transmission of the spatial location of resources from older to younger individuals leading to social inheritance. The accumulation of advantageous modifications of behavior in populations across generations may produce differential local adaptation between populations in socially learned traits, based on local environmental conditions and geography in much the same way as local adaptation through genetic differentiation does.

There are two non-mutually exclusive mechanisms by which socially learned behaviors can be transmitted to successive generations in a population. In his review, Whiten [7] puts forth the parallels between genetic and social inheritance, where the former encompasses genetic changes that spread throughout populations, and the latter pertains to the spread of socially learned behaviors over generations [8]. Genetic or epigenetic inheritance is based on DNA, RNA or protein materials present in the parental germ cells that are passed to the offspring when zygotes are formed. Social learning is transmitted independently from the germ line material through perception and acquisition of behaviors between individuals belonging to successive, yet overlapping generations. Genetic and social inheritance can thus function alone or interact and act simultaneously ([45,46,47,48]; see Figure 2 using oviposition site selection as an example).

There is evidence that most behaviors and behavioral variation between individuals have some genetic basis [49,50,51,52,53]. For example, several candidate loci were identified and associated with phenotypic variation for memorizing locations in the fly *Drosophila melanogaster* [54]. The identification of candidate loci paves the way for finding the genetic basis of complex behavioral traits, including spatial exploration ability and memory retention of spatial location (e.g., of suitable resources, including host plants for oviposition). Genetic variants with higher learning capacity and memory retention may thus become more numerous in successive generations, when there is positive selection for oviposition site selection (Figure 2). There is further evidence that learning ability itself has a genetic basis and that there is genetic variation in learning ability between individuals in various invertebrate, non-social taxa (reviewed in [54,55,56,57,58]). One gene whose allelic variation and expression is associated with differential learning rate and memory retention is the *foraging* gene (“*for*”), a pleiotropic gene that produces a cyclic GMP-dependent protein kinase (PKG), a protein involved in many regulatory functions, including energy homeostasis [59,60,61,62]. Although the exact function of *for* in learning (and social learning) remains to be understood [63], the existence of genetic variation for learning ability suggests that genetically “better” learners can proportionally increase in subsequent generations, for example if social learning of oviposition site selection from con-specifics is locally adaptive. 

Behaviors can also be transmitted epigenetically from parents to offspring, as was found for multiple behaviors and species [64,65,66]. For example, mice exposed to a neutral fruity odor while receiving a mild electric shock adopt a startle behavior later in life while only experiencing the odor, a behavior that is subsequently passed on to their children and grandchildren when sensing the odor without ever experiencing the shock [67,68]. These results pointed to the fixation of epigenetic variation affecting the expression of olfactory genes [67,69,70,71]. There is, to the best of our knowledge, no evidence yet for epigenetic transmission of spatial localization and memory of suitable resources, as depicted in our example of Figure 2, nor for other behaviors typically related to oviposition site selection in arthropods, such as transmission of preference for novel specific host plant species across generations [68,72]. It will be important to tease apart the contribution of the genome, epigenome, and social inheritance (described below), to understand how insects track and potentially adapt to rarefying suitable habitats through oviposition site selection behavior [73].

The second main inheritance mechanism, social inheritance, is based on social learning of behaviors between interacting individuals, such that learned behaviors can also be propagated without a genetic or epigenetic material basis across generations (Figure 1 and Figure 2). Social inheritance has so far mainly been observed in social vertebrates and more recently in social insects (e.g., [15]) and non-social insects (e.g., *Drosophila;* [23]). Social learning can increase local adaptation of individuals relying on socially acquired information by increasing their chance of finding a resource, or reducing the time or energetic cost these individuals need for finding and remembering the location of a resource, such as host plants for oviposition in a new suitable habitat (i.e., oviposition site selection; Figure 2A,B). Social learners may thus have overall quicker and/or more access to suitable resources for survival and reproduction compared to conspecifics that are not using or remembering social information. This, in turn, may lead to increased reliance on social information across generations (Figure 2C), whether socially acquired traits are transmitted over longer evolutionary times and multiple generations by culture or not.

Learning the location of a suitable plant for oviposition from a skilled con-specific may represent an important evolutionary advantage compared to non-social learning of host plant location. This is because non-social learners can be in a coevolutionary arms race (i.e., Red Queen dynamics) with their host plants, given that plants are under strong selection to avoid larval feeding using elusive traits for herbivorous arthropods (e.g., a similar shape and color as non-host plants, and distinct morphologies such as “butterfly egg mimicry” or apostatic selection) [35,74,75]. Social learners can thus avoid having to “reinvent the wheel” when it comes to finding suitable host plants by following, copying or imitating others. Two key aspects of social inheritance now need to be examined and tested both in the laboratory and in the field. First, it will be important to quantify to what extent social inheritance occurs throughout the diversity of evolving life, compared to genetic inheritance (all living species have DNA or RNA and cell division), including in non-social animals. Second, quantifying the adaptive value of social learning is of central importance (as depicted in steps A and B of Figure 2), whether socially acquired traits are transmitted over longer evolutionary times, or not. 

## 3. Relevance of Social Inheritance in Non-Social Arthropods

Socially acquired behaviors cause social inheritance only if they are transmitted over multiple generations. It is now timely to examine the extent of the transmission of socially acquired behaviors as a second inheritance system in nature (step 4 in Figure 1, Figure 2C). Small-brained, non-social invertebrates are particularly relevant to study, because they make up at least half of the species diversity on Earth [76,77]. The transmission of socially acquired behaviors across generations requires that individuals of different life stages or age groups live in contact with each other (Figure 1) [78]. For social inheritance to occur, generations must therefore be overlapping. This is indeed the case for eusocial species (i.e., with a clear reproductive division) that have overlapping generations by definition, but many non-social insects also have overlapping generations [78]. Furthermore, several insect taxa have a social population structure allowing the transmission of socially acquired behaviors over generations, through maternal, paternal and biparental care [79,80,81]. Maternal and biparental care takes the form of egg and/or offspring guarding, defense, nidification, and/or feeding facilitation or progressive provisioning and underpins the single most widespread form of sociality found in “non-eusocial” insects. These behaviors have been reported for >40 insect families belonging to 12 orders, as well as several non-insect arthropod groups, such as spiders, scorpions, opiliones, mites, chilopodes, and amphipod crustaceans [79]. Moreover, in a diverse array of mainly hemimetabolous arthropods, including treehoppers, true bugs, thrips, cockroaches and social spiders [82], mixed supercolonies of adults and immatures are found. While historically social inheritance has not actively been looked for in most insect taxa to date, the social structure of many insect species provides opportunities for transmission and inheritance of socially acquired behaviors far beyond the few documented cases in well-known, emblematic, social insects. 

## 4. Social Learning of Oviposition-Related Behavior from Con- and Hetero-Specifics

Research on social learning in non-social organisms is becoming a burgeoning field and progressively more evidence is being put forward. We focus on evidence for social learning involved in oviposition behavior (Table 1; but see [30,31,32,33] for social learning of foraging, mating, and other behaviors). The first step to show evidence of social learning is that a behavior is modified in response to the perception of a social cue (step 1 in Figure 1). As a large number of studies document the existence of step 1 in various non-social arthropods, we did not include these studies in Table 1 (e.g., [35,83,84,85,86,87,88,89,90,91,92,93]). Historically, most studies on oviposition-related behaviors have focused on parasitoid wasps (Hymenoptera) as model systems, where oviposition takes place in or on the body of another arthropod [94]. These studies were reviewed elsewhere [34] and we only cite a few representative case studies in Table 1. Many wasps use previous experiences with a hetero-specific (i.e., the host) during development or as adults as a social cue leading to a marked change in oviposition behavior compared to naive individuals (Table 1). Table 1 summarizes the evidence of 11 key studies focusing on social learning across 4 taxonomic orders within Arthropoda: the insect orders Hymenoptera (wasps), Diptera (flies), and Coleoptera (beetles) and the arachnid order Trombidiformes (mites). We thus see that modification of oviposition in response to earlier experience of social cues occurs in diverse arthropod orders and we expect many other non-social arthropods to use social learning, with a potential for social transmission and inheritance. 

Evidence for social learning of oviposition-related behaviors from con-specifics has been particularly well-documented in *Drosophila* flies (Table 1), where a typical experiment entails comparing fruit substrate preference for oviposition of flies with or without an occasion to observe “trained” congeners displaying a strong preference for a specific oviposition substrate. Training to develop a preference for a specific oviposition substrate (i.e., strawberry) is obtained by associating another substrate (i.e., banana) to an oviposition deterrent, such as quinine. Flies then develop a preference for another, simultaneously available, substrate (i.e., strawberry). Adult female flies further learn to interpret and use a wide variety of cues from con-specifics at different life stages when choosing an oviposition site. Visual cues, such as the presence of con-specific eggs and/or larvae on oviposition substrates, interactions with more experienced female demonstrators, as well olfactory cues produced by con-specifics have been shown to positively influence female oviposition decisions after the original cue has been removed. This suggests that the benefits of con-specific attraction in oviposition site selection may outweigh the costs of competition in the wild [85,86]. In the context of research on social learning in *Drosophila*, the large knowledge-base on cues used for oviposition site selection, as well as the documented evidence for social learning (Table 1), make it an excellent model for testing whether social learning of oviposition sites can be inherited socially. 

Acquiring social information from other species can be an efficient way to increase fitness. This is particularly true for non-social insects with limited access to information from con-specifics (such as for early dispersers, insects with small population sizes, and/or species with low con-specific encounter rates. Such species can use information from other species sharing aspects of their ecological niche to make nest choice decisions [95]. An interesting example of hetero-specific social learning can be found in the parasitic wasp *Trichogramma evanescens* [96]. Like its congener *T. brassicae*, this wasp uses the pheromones of its adult host, the butterfly *Pieris brassicae* to identify mated females that will subsequently lay eggs suitable for parasitism by the wasp. By using this information, the wasp will hitch-hike along for the ride to a new oviposition opportunity (i.e., the egg laying site of *P. brassicae*), but unlike *T. brassicae, T. evanescens* needs to learn through an oviposition experience that both host pheromones (to identify adult hosts) and hitch-hiking (towards host eggs) lead to a suitable oviposition site [96]. Several solitary bee species provide another example of social learning from hetero-specifics [95]. The cavity-nesting mason bees *Osmia caerulescens* and *O. leaiana* examine the nests of another congener, *O. bicornis,* for evidence of brood cell parasites. Though associative learning of nest site quality of congeners (using geometric symbols), *O. caerulescens* and *O. leaiana* preferred to start their own nest at sites associated with healthy nests of *O. bicornis* and rejected sites associated with brood cell parasites. This study is exceptional, because observations and experiments were conducted in the field using wild bees, although the possibility that nest selection behavior is innate and not due to social learning could not be ruled out completely [95].

**Table 1:** List of studies on non-social arthropods where social cue perception, social learning, and transmission of socially learned oviposition-related behaviors was quantified. Only studies that document social learning are included (i.e., from step 2 of Figure 1 onwards), as there is a large body of literature covering cue perception (i.e., step 1 of Figure 1). The table includes the species, the order (Diptera = D, Hymenoptera = H, C = Coleoptera, Trombidiformes = T), the type of social cue and the behavior under study, con- (c) or hetero- (h) specific social learning, the steps towards social inheritance (as in Figure 1) and if effects on fitness were quantified in the study. Studies concerned with foraging, mating, host finding and other behaviors, including in non-insect invertebrates, have been discussed elsewhere [30,31,32,33,34].

Species	Order	Social Cue	Behavior	Learning from con- (c) or Hetero- (h) Specifics	Step Towards Social Inheritance	Fitness Tested	Reference
*D. melanogaster*	D	Experienced females with preferred oviposition site	Site selection	c	1, 2, 3	y	[25]
*D. melanogaster*	D	Parasitoid presence (i.e., threat to offspring survival)	Clutch size	c	1, 2	y	[21]
*Drosophila* spp.	D	Parasitoid presence (i.e., threat to offspring survival)	Clutch size	c + h	1, 2, 3	y	[22]
*D. melanogaster*	D	Mated females	Site selection	c	1, 2	y	[97]
*Leptopilina boulardi*	H	Host insect	Site selection	h	1, 2	n	[98]
*Necremnus tutae*	H	Host insect and plant species	Host species preference	h	1, 2	n	[99]
*Osmia* sp.*	H	Nest site parasitism	Site selection	h	1, 2	n	[95]
*Trichogramma evanescens*	H	Host adult and eggs	Phoresy to oviposition substrate	h	1, 2	n	[96]
*Anisopteromalus calandrae*	H	Host insect	Host preference + host-finding + parasitism rates	h	1, 2	y	[100]
*Phratora vulgatissima*	C	Adult females	Distance between clutches	c	1, 2	y	[101]
*Tetranychus urticae, T. kanzawai*	T	Predator	Site selection (leaf surface vs web)	h	1, 2	n	[102]

* Tested under field conditions.

The value of social information from hetero-specifics has also been studied in *Drosophila*. Particularly noteworthy is the flow of social information in the genus *Drosophila* related to the presence of a parasitoid observed by Kacsoh and co-authors [22]. The divergence in social cues that evolved between different species led to the formation of species-specific communication patterns (referred to as “dialects”). The magnitude of divergence in species-specific communication patterns was found to be correlated with the phylogenetic distance between species. Kacsoh et al. [22] exploited this system to test whether the degree of hetero-specific social information transfer between *Drosophila* species was related to their relative phylogenetic distance, hypothesizing that phylogenetically close species are more successful in sharing social information. Similar to earlier experiments by Kacsoh et al. [21] (Figure 1), *Drosophila* females were presented with visual cues of parasitic wasps that led to a reduction in the number of eggs laid. When the experienced fly belonged to a different species, Kacsoh et al. [22] observed the same decrease in number of eggs laid. While closely related *Drosophila* species were able to efficiently communicate information about the presence of the parasitoid, species that were phylogenetically more distant had limited to no communication abilities. Interestingly, multi-species communities enhanced inter-specific communication, allowing *Drosophila* to learn multiple dialects. This indicates a degree of plasticity in learning abilities that could be adaptive in nature when *Drosophila* species occur in sympatry [22]. This study represents a rare empirical test for socially learned behaviors can be transmitted to others in non-social invertebrates (i.e., up to step 3 in Figure 1).

Evidence for social learning has been based on at least three experimental setups: some studies compare the behavior of individuals before (test a), during (test b) and after (test c) experiencing the social cue. Evidence for social learning becomes apparent when the behaviors observed in tests b and c are similar, but different from the behavior displayed in test a. Another, better design, takes ageing (and its potential confounding effect) into account by comparing groups of naive individuals with experienced individuals (that had an earlier experience with the social cue) of similar age. The behaviors of the naive and experienced groups should differ in the absence of the social cue to show evidence of social learning in the experienced group. A third setup consists of associating a social cue to another cue (that does not need to be social, i.e., color, symbols etc.), and comparing the behavior of a group of naive individuals with a group that experienced the social *and* the associated cue, in the presence of only the associated cue. Evidence for social learning is then based on a significant difference in behavior between the two groups in the presence of the associated (but not the social) cue, for the experienced group. These experimental set-ups, when carefully designed, allow to discriminate beyond any doubt socially learned behaviors from behaviors that are innate or learned as consequence of interactions with abiotic cues. As such, they provide excellent opportunities to study social learning, transmission, and inheritance of oviposition-related (and other) behaviors in a wide range of non-social arthropods. In light of the accumulating evidence for widespread social learning, these experimental designs can greatly contribute to our understanding of the role of social learning in evolution.

Evidence for hetero-specific social learning has also been found for behaviors other than oviposition. Social learning in non-social arthropods was first reported in a cricket, *Nemobius sylvestris*, that changed its predator avoidance behavior based on observations, and memory of such observations, of either predator presence (spiders) or of congener crickets that had already experienced the presence of spiders [26]. Hetero-specific social information can thus also be transmitted from experienced to naive crickets [26], which can decrease predation risk. Hetero-specific social information was also found to increase the efficiency of locating food sources [103,104,105,106]. Although social information from hetero-specifics is ubiquitous, it can be challenging to decode, for example because the cue may have had a different original meaning or purpose than what is interpreted by the receiving species [107,108,109,110].

## 5. The Adaptive Value of Social Learning

Social learning is an important mechanism in evolution even when transmission of socially acquired behaviors is limited to a few generations within a season, such that social inheritance will not be maintained over long evolutionary times (step 4 in Figure 1). Indeed, we suggest that building expertise during a lifetime by social experiences can increase the adaptation rate of populations that are using and memorizing social information, for example for the spatial location of essential resources, even if every adult individual dies at the end of the reproductive season. This is, for example, because social information allows individuals to avoid unfavorable oviposition sites, to reach an oviposition site earlier or at lower exploratory costs, compared to individuals that explore and spatially navigate without this information. In this regard, most current evidence for social learning, including in non-social insects, concerns behaviors such as foraging and host location, which are based on resources that vary rapidly in space and time notably due to seasonal changes. Related social information is thus of ephemeral relevance as well and it needs to be updated constantly, suppressing the emergence of any form of longer-term social inheritance. Rupture of socially transmitted behaviors can also take place because most representatives of insect populations die seasonally, for example during winter in temperate regions. In the latter case, social information about resources can be acquired and exchanged socially *de novo* at the beginning of the new reproductive season each year, starting from newly emerged naive individuals in spring that learn about resource distribution in their surrounding environment.

The adaptive value of learned behaviors is documented in some vertebrates [4,5], but experimental evidence for the adaptive value of socially learned behaviors in ecologically relevant conditions currently remains unquantified for the vast majority of living taxa [17,111], including non-social insects [112]. Social learning can increase the fitness of individuals and as such be under positive selection in rapidly changing environments. Yet, this is not necessarily the case as negative effects on fitness were documented from partially or incorrectly interpreted social cues that caused increased energy expenditure in basic tasks, such as foraging [108]. The costs associated with social learning, including energetic costs and time constraints, and the environmental parameters under which social learning becomes adaptive, have been explored both experimentally [113] and through modeling work [114,115]. These studies have revealed that social learning is not necessarily adaptive under all conditions and that learning can lead to evolutionary traps under rapidly changing environmental conditions [116]. 

A study with *D. melanogaster* convincingly suggested that social learning has adaptive value also in the context of oviposition-related behaviors in non-social insects [21]. Here, the authors exposed ovipositing *D. melanogaster* females to a parasitoid wasp that lays eggs inside *D. melanogaster* larvae, which are subsequently consumed from the inside out by the developing parasitoid. Having been faced with a serious threat to the survival of their offspring [117], female *D. melanogaster* reduced the number of eggs laid in the next clutch [118]. When a parasitoid-experienced fly was then observed by a naive female fly, the latter also reduced her clutch size, even though the original social cue, the wasp, was no longer present [21]. Within an ecological context, reducing egg numbers in the face of an immediate threat to offspring survival can have a clear adaptive value, also under natural conditions. Indeed the wasp species used in this study actively searches for host patches in the environment [119,120], where mating, oviposition or feeding *Drosophila* larvae generate perceivable olfactory cues for the wasp [121]. It remains to be tested whether social learning in *D. melanogaster* females can be transmitted from one generation to the next (as was found in [23]).

## 6. Conclusions

Perception of social cues, social learning and transmission are the stepping stones towards social inheritance. While perception of social cues is now well known to induce behavioral changes in multiple arthropods (e.g., [35,83,84,85,86,87,88,89,90,91,92,93]), we need to increase our understanding of social learning in non-social arthropods and determine its prevalence, both in the laboratory and in the field. Due to its inherent link to fitness, oviposition site selection offers unparalleled opportunities to study social learning and transmission, also in systems other than *Drosophila.* The increasing number of studies on social learning in non-social arthropods (see Table 1) offer promising possibilities for empirical tests of social transmission and inheritance.

## Data Availability

Not applicable.
